# Incidence, Risk Factors, and Outcomes of Severe Hypoxemia After Cardiac Surgery

**DOI:** 10.3389/fcvm.2022.934533

**Published:** 2022-06-28

**Authors:** Dashuai Wang, Xiangchao Ding, Yunshu Su, Peiwen Yang, Xinling Du, Manda Sun, Xiaofan Huang, Zhang Yue, Fuqiang Sun, Fei Xie, Chao Liu

**Affiliations:** ^1^Department of Cardiovascular Surgery, Union Hospital, Tongji Medical College, Huazhong University of Science and Technology, Wuhan, China; ^2^Department of Cardiovascular Surgery, The First Affiliated Hospital of Zhengzhou University, Zhengzhou, China; ^3^Department of Thoracic Surgery, Renmin Hospital of Wuhan University, Wuhan, China; ^4^China Medical University-The Queen’s University of Belfast Joint College, China Medical University, Shenyang, China

**Keywords:** hypoxemia, cardiac surgery, risk factor, prediction model, nomogram

## Abstract

**Background:**

Hypoxemia is common in patients undergoing cardiac surgery, however, few studies about severe hypoxemia (SH) after cardiac surgery exist. The objectives of this study were to clarify the incidence, risk factors, and outcomes of SH after cardiac surgery.

**Methods:**

Patients undergoing cardiac surgery from 2016 to 2019 in a single center were enrolled and were divided into two groups based on whether postoperative SH developed. Independent risk factors for SH were identified by univariate and multivariate analysis. Model selection statistics were applied to help determine the most parsimonious final model.

**Results:**

Severe hypoxemia developed in 222 of the 5,323 included patients (4.2%), was associated with poorer clinical outcomes. Six independent risk factors for SH after cardiac surgery were identified by multivariate analysis, such as surgical types, white blood cell (WBC) count, body mass index (BMI), serum albumin, cardiopulmonary bypass (CPB) time, and intraoperative transfusion of red blood cells (RBCs). After comprehensively considering the discrimination, calibration, and simplicity, the most appropriate and parsimonious model was finally established using four predictors, such as WBC count, BMI, CPB time, and intraoperative transfusion of RBCs. A nomogram and a web-based risk calculator based on the final model were constructed to facilitate clinical practice. Patients were stratified into three risk groups based on the nomogram and clinical practice.

**Conclusion:**

Severe hypoxemia was common after cardiac surgery and was associated with poorer clinical outcomes. A parsimonious final model with good discrimination, calibration, and clinical utility was constructed, which may be helpful for personalized risk assessment and targeted intervention.

## Introduction

Postoperative hypoxemia is one of the most common complications after cardiac surgery, and is associated with a higher risk of morbidity and mortality (1–3). Due to different definitions and populations in various studies, the incidence of hypoxemia varies widely in the literature, up to 84% (1).

Several studies focused on predictors for severe hypoxemia (SH) after cardiac surgery have been carried out and some significant risk factors have been identified, such as body mass index (BMI) and cardiopulmonary bypass (CPB) time (4–6). However, most of these studies were conducted in patients undergoing surgery for acute type A aortic dissection due to its ultrahigh prevalence. Moreover, the majority of the previous studies reported in the literature were based on small sample sizes, and there is still a lack of a credible large sample study in this field. In addition, none of those previous studies developed a reasonable online tool, such as a web-based risk calculator for SH after cardiac surgery, which may be more convenient for modern clinical work. Therefore, our understanding of the predictors for SH following cardiac surgery remains limited, and developing a risk prediction model is still an urgent priority.

The objectives of this study were first to clarify the incidence and predictors of SH after cardiac surgery and to develop a parsimonious risk prediction model, and second to clarify the relationship between SH and clinical outcomes.

## Materials and Methods

### Ethics Statement

The study was conducted according to the Declaration of Helsinki’s ethical principles. Study approval was granted by the Ethics Committee of Tongji Medical College of Huazhong University of Science and Technology (IORG No. IORG0003571). As this was a retrospective observational study, written informed consent was waived.

### Study Population

Consecutive adults (older than 18 years) undergoing cardiac surgery in a tertiary medical center between January 2016 and December 2019 were enrolled. Those who died intraoperatively of immunodeficiency, immunosuppression, or organ transplantation, as well as those with incomplete medical records, were excluded from the current study.

### Data Extraction

Clinical data collection was completed through the electronic medical records management system of our hospital. Baseline characteristics collected and compared in our analysis included patients’ age, sex, BMI, smoking history, drinking history, diabetes mellitus, hypertension, chronic obstructive pulmonary disease, cerebrovascular disease, peripheral vascular disease, atrial fibrillation, gastrointestinal tract disease, renal insufficiency, previous cardiac surgery, general surgical history, New York Heart Association (NYHA) class, pericardial effusion, pulmonary artery hypertension, the diameter of the left and right atria and ventricles, left ventricular ejection fraction, counts of white blood cell (WBC), red blood cell (RBC) and platelet, hemoglobin, serum creatinine, albumin, and globulin. Intraoperative factors included surgical types, CPB time, aortic cross-clamp time, and transfusion of RBCs.

### Endpoints and Definitions

The primary endpoint was SH after cardiac surgery in this study. The arterial blood gas measurements were routinely performed postoperatively using a blood gas analyzer (Radiometer, ABL800PLEX, Denmark). The oxygenation index was calculated from arterial oxygen partial pressure and inspired oxygen concentration (PaO_2_-FiO_2_). In this study, we defined SH as PaO_2_/FiO_2_ ≤ 100 mmHg based on previous reports in the literature and the diagnosis of acute respiratory distress syndrome in the Berlin definition (1, 7). SH was diagnosed based on the worst values of the recorded PaO_2_/FiO_2_ ratios within the first 24 h postoperatively, and patients were classified into the SH group and the non-SH group according to whether SH occurred within the first 24 h after cardiac surgery.

The secondary endpoints included the duration of mechanical ventilation, pneumonia, reintubation, tracheostomy, readmission to the intensive care unit (ICU), the duration of ICU stay, the duration of hospital stay, and in-hospital mortality.

### Statistical Analysis

A statistical analysis was conducted using R software (version 4.0.5)^1^ and SPSS (IBM SPSS Statistics 26.0, SPSS Inc., Chicago, IL). A two-tailed *p*-value of less than 0.05 was considered statistically significant.

To test whether continuous variables were distributed normally, Kolmogorov–Smirnov tests were used. Continuous variables were expressed as means ± standard deviations (SDs) for normally distributed variables and as medians with interquartile ranges (IQRs) for skewed variables. Categorical variables were presented as counts and percentages. A univariate analysis was conducted first to screen for potential risk factors. Student’s *t*-test and Mann–Whitney *U*-test were applied to continuous variables. The chi-square test and Fisher’s exact test were applied to categorical variables. Then, a forward stepwise multivariate logistic regression analysis was conducted to identify independent risk factors for SH after cardiac surgery. An odds ratio (OR) with a 95% confidence interval (*CI*) was calculated. Model selection statistics were applied to help determine the most parsimonious final model considering the discrimination, calibration, and simplicity. A nomogram and a web-based risk calculator were constructed based on the final model.

A bootstrap method with 1,000 replications was used for internal validation. A Hosmer–Lemeshow goodness-of-fit test and a calibration plot were used to evaluate calibration. The area under the receiver operating characteristic (ROC) curve (AUC) was used to evaluate discrimination. A decision curve analysis was used to evaluate clinical utility.

## Results

### Demographic Characteristics

A total of 5,323 cases met the inclusion criteria and were included in this study, 51.4% were men. The average age of these patients was 51.18 years and the BMI was 23.35 kg/m^2^. The incidence of SH within the first 24 h after cardiac surgery was 4.2% (222/5,323).

In this study population, there were multiple underlying conditions and comorbidities. Smoking history existed in 29.1% of these patients, drinking history in 21.7%, hypertension in 30.1%, diabetes mellitus in 8.2%, chronic obstructive pulmonary disease in 10.5%, atrial fibrillation in 17.1%, renal insufficiency in 9.8%, pulmonary artery hypertension in 26.1%, pericardial effusion in 14.2%, gastrointestinal tract disease in 8.4%, cardiac surgery history in 7.1%, and general surgery history in 28.1% of the patients.

Of the 5,323 operations, 54% were performed for isolated valve surgery, 11% for isolated coronary artery bypass grafting, 9% for mixed valve and coronary surgery, 17% for aortic surgery, and 9% for other types of surgery. The median CPB time and aortic cross-clamp time were, respectively, 105 (78, 142) and 69 (46, 95) min, and the intraoperative transfusion of RBCs was 1 (1, 3) units.

### Identification of Independent Risk Factors

A univariate analysis was first performed to screen possible risk factors for SH after cardiac surgery (Table 1). Those factors with a *p*-value less than 0.1 or deemed clinically relevant were further analyzed by multivariate analysis, such as sex, age, BMI, smoking history, drinking history, hypertension, chronic obstructive pulmonary disease, cerebrovascular disease, peripheral vascular disease, atrial fibrillation, renal insufficiency, general surgery history, NYHA class, pulmonary artery hypertension, pericardial effusion, diameter of the left atrium, diameter of the right atrium, WBC count, hemoglobin, platelet count, serum albumin, serum globulin, serum creatinine, surgical types, CPB time, and intraoperative transfusion of RBCs. After multivariate analysis, six independent risk factors for SH after cardiac surgery were identified, such as aortic surgery, WBC, BMI, serum albumin, CPB time, and transfusion of RBCs (Supplementary Table 1). All regression analyses were checked for multicollinearity.

**TABLE 1:** The univariate analysis of possible risk factors for SH after cardiac surgery.

Characteristics	Without SH *n* = 5,101 (%)	With SH *n* = 222 (%)	*P*-value
**Demographics**			
Male	2,827 (55.4)	174 (78.4)	<0.001
Age (years)	51.18 ± 12.98	51.00 ± 11.40	0.821
Height (cm)	164.80 ± 8.00	168.94 ± 7.86	<0.001
Weight (kg)	63.29 ± 11.64	76.81 ± 14.10	<0.001
Body mass index (kg/m^2^)	23.20 ± 3.31	26.76 ± 3.64	<0.001
Smoking history	1,445 (28.3)	103 (46.4)	<0.001
Drinking history	1,072 (21.0)	82 (36.9)	<0.001
**Underlying conditions**			
Hypertension	1,464 (28.7)	140 (63.1)	<0.001
Diabetes mellitus	420 (8.2)	18 (8.1)	0.947
Chronic obstructive pulmonary disease	542 (10.6)	15 (6.8)	0.065
Cerebrovascular disease	1,759 (34.5)	53 (23.9)	0.001
Peripheral vascular disease	2,211 (43.3)	47 (21.2)	<0.001
Atrial fibrillation	897 (17.6)	15 (6.8)	<0.001
Renal insufficiency	432 (8.5)	92 (41.4)	<0.001
Gastrointestinal tract disease	433 (8.5)	14 (6.3)	0.251
Pulmonary edema	244 (4.8)	9 (4.1)	0.617
Cardiac surgery history	361 (7.1)	15 (6.8)	0.855
General surgery history	1,450 (28.4)	47 (21.2)	0.019
NYHA class III-IV	851 (16.7)	25 (11.3)	0.033
Pulmonary artery hypertension	1,367 (26.8)	23 (10.4)	<0.001
Pericardial effusion	699 (13.7)	57 (25.7)	<0.001
Diameter of the left atrium (cm)	4.2 (3.6, 5.0)	3.8 (3.4, 4.3)	<0.001
Diameter of the left ventricle (cm)	5.0 (4.5, 5.7)	4.9 (4.5, 5.5)	0.120
Diameter of the right atrium (cm)	3.8 (3.5, 4.4)	3.8 (3.5, 4.2)	0.019
Diameter of the right ventricle (cm)	3.6 (3.3, 4.0)	3.7 (3.4, 4.0)	0.450
Left ventricular ejection fraction (%)	62 (58, 66)	61 (60, 65)	0.174
**Laboratory values**			
White blood cell count (×10^9^/L)	5.73 (4.75, 7.01)	9.74 (7.10, 12.70)	<0.001
Red blood cell count (×10^12^/L)	4.27 (3.92, 4.63)	4.35 (3.90, 4.69)	0.218
Hemoglobin (g/l)	129 (118, 140)	133 (120, 143)	0.040
Platelet count (×10^9^/L)	180 (145, 221)	167 (127, 206)	<0.001
Serum albumin (g/L)	40.5 (38.0, 42.8)	38.6 (35.9, 41.3)	<0.001
Serum globulin (g/L)	24.3 (21.7, 27.2)	25.0 (22.2, 28.2)	0.028
Serum creatinine (μmol/L)	71.6 (60.7, 84.8)	82.9 (68.4, 112.3)	<0.001
Surgical types			<0.001
Isolated valve surgery	2,861 (56.1)	36 (16.2)	
Isolated coronary artery bypass grafting	580 (11.4)	11 (5.0)	
Mixed valve and coronary artery bypass grafting	468 (9.2)	13 (5.8)	
Aortic surgery	746 (14.6)	158 (71.2)	
Other types	446 (8.7)	4 (1.8)	
Cardiopulmonary bypass time (minutes)	103 (77, 138)	200 (146, 260)	<0.001
Aortic cross clamp time (minutes)	67 (46, 92)	113 (86, 146)	<0.001
Transfusion of red blood cells (units)	1 (1, 3)	4 (3, 7)	<0.001

*NYHA, New York Heart Association; SH, severe hypoxemia.*

### Model Construction

**FIGURE 1:**
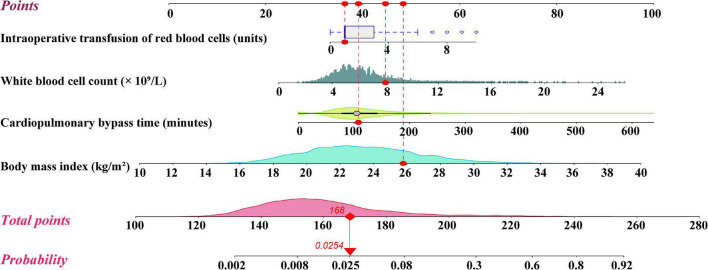
Nomogram for the prediction of severe hypoxemia after cardiac surgery.

The logistic regression model (model 1) constructed using the six predictors above showed good discrimination (AUC = 0.912, 95% CI, [0.893–0.932]) and calibration (Hosmer–Lemeshow χ^2^-value = 5.615, *p* = 0.690). On the basis of model 1, we constructed multiple models by reducing the variables in the model to identify the most parsimonious model without losing discrimination and calibration (Supplementary Table 2–11, models 2–11). The comparison of statistics concerning discrimination and calibration of these models are presented in Table 2. After comprehensive comparison and screening, we finally selected model 5 as the most appropriate and parsimonious model, which indicated excellent discriminative ability (AUC = 0.911, 95% CI, [0.891–0.930]) and calibration (Hosmer–Lemeshow χ^2^-value = 8.772, *p* = 0.362) at the same time. Four independent risk factors were included in model 5, such as WBC, BMI, CPB time, and transfusion of RBCs (Supplementary Table 5). Based on the four predictors in model 5 and the logistic rule, a visual nomogram was constructed used to facilitate the prediction of SH after cardiac surgery (Figure 1). Based on the regression coefficient of each variable, we scaled their scores to 0–100 points to reflect their relative importance.

**TABLE 2:** Comparison of the multiple logistic regression models for SH after cardiac surgery.

Models	Number of predictors	Included predictors	AUC (95% CI)	Hosmer-lemeshow goodness-of-fit test	
				χ^2^-value	*P*-value
Model 1	6	BMI, WBC, albumin, surgical types, CPB time, transfusion of RBCs	0.912 (0.893–0.932)	5.615	0.690
Model 2	5	BMI, WBC, albumin, CPB time, transfusion of RBCs	0.911 (0.891–0.931)	8.811	0.359
Model 3	4	BMI, albumin, CPB time, transfusion of RBCs	0.900 (0.880–0.920)	10.760	0.216
Model 4	4	WBC, albumin, CPB time, transfusion of RBCs	0.875 (0.848–0.901)	4.954	0.763
Model 5	4	BMI, WBC, CPB time, transfusion of RBCs	0.911 (0.891–0.930)	8.772	0.362
Model 6	4	BMI, WBC, albumin, transfusion of RBCs	0.901 (0.880–0.923)	6.227	0.622
Model 7	4	BMI, WBC, albumin, CPB time	0.901 (0.878–0.923)	8.335	0.401
Model 8	3	BMI, CPB time, transfusion of RBCs	0.897 (0.878–0.917)	11.396	0.180
Model 9	3	WBC, CPB time, transfusion of RBCs	0.874 (0.848–0.901)	9.397	0.310
Model 10	3	BMI, WBC, transfusion of RBCs	0.901 (0.880–0.922)	8.772	0.362
Model 11	3	BMI, WBC, CPB time	0.899 (0.876–0.922)	11.149	0.193

*AUC, area under the receiver operating characteristic curve; BMI, body mass index; CI, confidence interval; CPB, cardiopulmonary bypass; RBC, red blood cell; SH, severe hypoxemia; WBC, white blood cell.*

Using the nomogram, the risk of SH in patients undergoing cardiac surgery can be easily predicted by adding the points of the four predictors. Patients who have a higher BMI, higher WBC count, longer CPB time, and more intraoperative transfusion of RBCs can have higher total points and a resultant higher risk of SH. A specific patient is shown in Figure 1 to show the use of the nomogram. In addition, to better adapt to modern clinical work, we created a web-based risk calculator for SH after cardiac surgery, which is available online.^2^

### Model Assessment and Internal Validation

**FIGURE 2:**
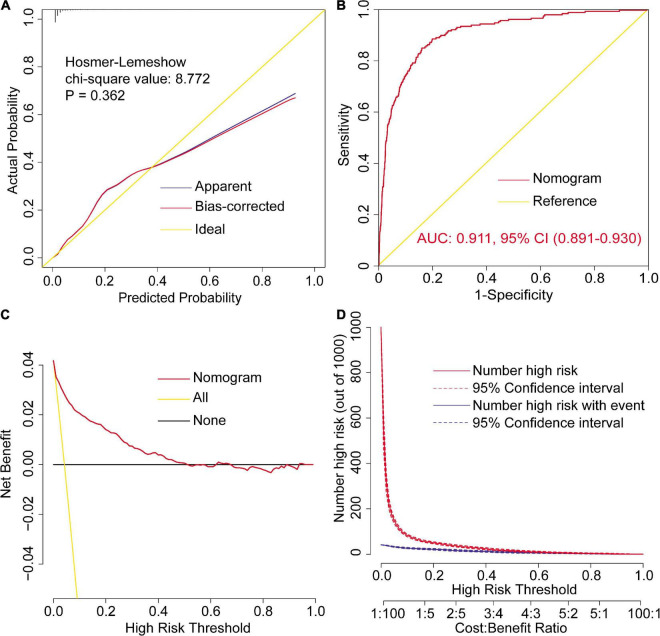
Assessment of the final model for severe hypoxemia after cardiac surgery. Calibration plots and goodness-of-fit test **(A)**, ROC curves and the AUC **(B)**, decision curve **(C)**, and clinical impact curves **(D)**. AUC, area under the receiver operating characteristic curve; CI, confidence interval; ROC, receiver operating characteristic curve.

In addition to the goodness-of-fit test, the final nomogram model was well calibrated by visual inspection of the calibration plots (Figure 2A). The results of the internal validation by bootstrapping with 1,000 resamples showed that the final model performed well. By plotting the ROC curve and calculating the AUC, the nomogram model indicated excellent discriminative ability (Figure 2B). By the decision curve analysis, the nomogram model showed remarkable clinical usefulness (Figures 2C,D). The decision and clinical impact curves indicated that compared with the “treat-none” strategy, patients could obtain more clinical net benefits among the thresholds of below 0.5 when using the nomogram; and compared with the “treat-all” strategy, patients could obtain more clinical net benefits among almost the whole range of threshold probabilities.

**FIGURE 3:**
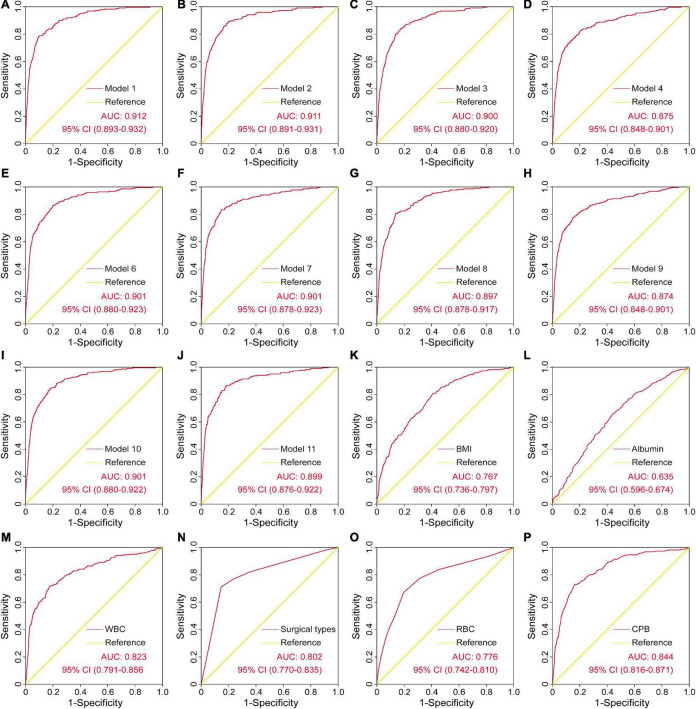
The ROC curves and the AUCs of the other ten models and the six independent risk factors for severe hypoxemia after cardiac surgery. AUC, area under the receiver operating characteristic curve; CI, confidence interval; ROC, receiver operating characteristic curve.

The ROC curves and the AUCs of the other ten models mentioned above were also plotted and calculated (Figures 3A–J). As can be clearly seen, the AUC of the final model was almost equal to the AUCs of model 1 and model 2, but was relatively greater than the other models. However, the number of variables in the final model was reduced to four from the original six independent risk factors, which may significantly reduce the model complexity and facilitate clinical application. In addition, the ROC curves and the AUCs of the six independent risk factors for SH after cardiac surgery were plotted and calculated (Figures 3K–P).

### Risk Stratification

To facilitate clinical application, a risk stratification was performed on the basis of the nomogram model for SH after cardiac surgery and clinical practice (Table 3). Predicted probabilities of 0.05 and 0.1 were selected as the cutoff values of low-, medium-, and high-risk groups, corresponding to the scores of < 179, 179–189, and > 189 points on the nomogram model. The consistency of the predicted and observed probabilities within each risk interval and the significant differences in different risk intervals also showed the good fit of the model and the rationality of the risk interval division. In this study, 85.1% of the patients were stratified into the low-risk group, 6.6% in the medium-risk group, and 8.3% in the high-risk group.

**TABLE 3:** Risk intervals of SH based on the nomogram and clinical practice.

Risk intervals	Low risk (<179 points)	Medium risk (179–189 points)	High risk (>189 points)
Predicted risk interval (%)	<5	5–10	>10
Predicted probability, % (95% CI)	1.3 (1.2–1.3)	6.9 (6.8–7.1)	31.7 (29.7–33.6)
Observed probability, % (95% CI)	1.1 (0.8–1.4)	8.9 (5.9–11.9)	32.3 (27.9–36.7)
No. of patients (%)	4,531 (85.1)	349 (6.6)	443 (8.3)

*CI, confidence interval; SH, severe hypoxemia.*

### Clinical Outcomes

Patients included in this study had an overall mortality rate of 3.1% (222/5,323), with a rate of 2.6% in patients without SH vs. 15.8% in those with SH (*p* < 0.001). In addition, the durations of mechanical ventilation, ICU stay, and hospital stay were significantly longer in patients with SH, and the rates of postoperative pneumonia, reintubation, tracheostomy, and readmission to the ICU were significantly higher compared to patients without SH (Table 4).

**TABLE 4:** Postoperative variables in patients with and without SH after cardiac surgery.

Variables	All cases *n* = 5,323 (%)	Without SH *n* = 5,101 (%)	With SH *n* = 222 (%)	*P*-value
Mechanical ventilation (hours)	22.6 (18.5, 43.6)	22.2 (18.2, 42.3)	90.1 (49.5, 170.2)	<0.001
Pneumonia	530 (10.0)	424 (8.3)	106 (47.7)	<0.001
Reintubation	247 (4.6)	210 (4.1)	37 (16.7)	<0.001
Tracheostomy	128 (2.4)	91 (1.8)	37 (16.7)	<0.001
Readmission to ICU	207 (3.9)	175 (3.4)	32 (14.4)	<0.001
ICU stay (hours)	68.3 (44.7, 112.6)	67.5 (44.5, 108.2)	202.9 (116.7, 315.7)	<0.001
Hospital stay (days)	15 (11, 19)	14 (11, 19)	22 (17, 30)	<0.001
Mortality	167 (3.1)	132 (2.6)	35 (15.8)	<0.001

*ICU, intensive care unit; SH, severe hypoxemia.*

## Discussion

Hypoxemia has been considered to be associated with a higher risk of poorer clinical outcomes after cardiac surgery (1–3), which was reconfirmed by this study. The incidence rate varied widely in previous reports according to different definitions and surgical populations (1, 5, 6, 8, 9). In the current study, the incidence rate of SH after cardiac surgery was 4.2%, and the mortality rate was 3.1%. However, the probabilities of in-hospital mortality and several other poor clinical outcomes were significantly higher in patients with SH, highlighting the need to explore significant risk factors and develop an authentic prediction model for SH after cardiac surgery.

Using clinical data from 5,323 patients in a single institution, we attempted to explore the predictors and develop a parsimonious prediction model for SH after cardiac surgery in this study. By univariate and multivariate analyses, a total of six significant risk factors associated with the occurrence of SH were identified, such as aortic surgery, WBC, BMI, serum albumin, CPB time, and transfusion of RBCs. After comprehensive comparison and screening, a final model with excellent discriminative ability, calibration, and clinical utility was considered to be the most appropriate and parsimonious model, which included four independent risk factors (WBC, BMI, CPB time, and transfusion of RBCs). A nomogram and a web-based risk calculator predicting the risk of SH after cardiac surgery was then established on the basis of the final model. Ultimately, three risk groups were divided based on the nomogram model and clinical practice. To our knowledge, this is currently the largest study in this field and the first attempt to construct a nomogram and a web-based risk calculator for SH after cardiac surgery worldwide, which may have certain guiding significance for clinical work.

A correlation between BMI and postoperative hypoxemia has been reported following various surgeries (3–5, 9–13), which was consistent with the results of the current study. In our analysis, BMI contributed the largest range of weight among all the predictors, and BMI alone showed moderate discrimination ability for SH after cardiac surgery (AUC = 0.767). Ranucci et al. conducted a large retrospective single-center study in patients undergoing cardiac operations with CPB to investigate the incidence, predictors, and outcomes associated with hypoxemia (3). They found that 30.6% of the patients developed hypoxemia and a significant inverse correlation between BMI and PaO_2_/FiO_2_ ratio was observed. The risk of hypoxemia increased by 1.7-fold per each incremental BMI class and BMI > 30 kg/m^2^ was associated with a 2.4-fold increased risk of postoperative hypoxemia. They also discovered a U-shaped relationship between BMI and ICU stay, with longer ICU stays in moderate-morbid obese and underweight patients. Zhou et al. conducted a single-center retrospective study to investigate independent risk factors for hypoxemia in patients undergoing surgery for acute type A aortic dissection, finding that patients with SH exhibited a remarkably higher BMI, and BMI was identified as an independent risk factor in the multivariate analysis. They illustrated that having a higher BMI was linked to the development of SH because obese patients often showed a decreased total lung capacity, functional residual capacity, expiratory reserve volume, and vital capacity. Moreover, obesity was associated with abdominal distention, decreased chest wall compliance, and increased respiratory resistance (14). In addition, the increase in chronic inflammatory response and oxidative stress has been reported to be associated with cell membrane damage and lung injury, which may provide new ideas for the treatment of hypoxemia (4, 12).

White blood cell was identified as a significant predictor for SH in the multivariate analysis and itself can exhibit a good discriminative ability (AUC = 0.823). This was consistent with some previous reports (6, 8). Liu et al. conducted a retrospective study in patients undergoing surgical repair of acute type A aortic dissection to identify the predictors for postoperative hypoxemia, finding that the incidence of hypoxemia was 30% and patients with hypoxemia had significantly longer durations of ventilation, ICU stay, and hospital stay (8). Preoperative WBC > 15,000/μl was identified as an independent risk factor for postoperative hypoxemia in their analysis, associated with a 9.8-fold increased risk. Ge et al. conducted a retrospective study in patients undergoing acute aortic dissection surgery to develop and validate a regression model to predict postoperative hypoxemia (8). After a purposeful selection procedure, WBC count was identified as an independent risk factor for postoperative hypoxemia, showing a positive correlation relationship. The association between higher WBC and an increased risk of hypoxemia may be partially explained by systemic inflammatory response. As one of the important biomarkers, higher WBC count reflected more intense inflammatory responses, which may result in respiratory dysfunction and hypoxemia (15, 16). Duan et al. suggested that the serum level of C-reactive protein was independently associated with preoperative hypoxemia, which was a sensitive and non-specific inflammatory marker of acute phase reactant. Recent attention to the relationship between inflammation and hypoxemia may provide new and enlightening insights (17). In addition, the use of some drugs, such as ulinastatin may produce significant protective effects (15).

Cardiopulmonary bypass time as an independent risk factor for hypoxemia has also been reported in previous studies (6, 11), which was also identified in our analysis. CPB time exhibited the best discriminative ability among the six independent risk factors, with an AUC of 0.844. Szeles et al. conducted an observational transversal study to identify predictive factors for SH in patients undergoing myocardial revascularization (11). They found that the application of CPB was associated with an increased risk of SH, and the risk increased to 3.1 times in patients with CPB greater than 120 min. In the findings of Wang et al., circulatory arrest time was independently associated with postoperative hypoxemia (18). Similar results were also obtained by Liu et al., who found that deep hypothermic circulatory arrest time > 25 min was an independent risk factor for postoperative hypoxemia, associated with a 3.3-fold increased risk (8). CPB can lead to systematic inflammatory responses, activate various cytokines and inflammatory mediators, and weaken immune responses (19, 20). Although progress has been made in CPB technique these years, CPB is still a non-physiological circulation, which may significantly alter the perfusion of peripheral tissues. To improve the weaknesses and deficiencies of traditional CPB, minimal invasive extracorporeal circulation has been introduced these years and the concept of “more physiologic” cardiac surgery has been proposed (21, 22). In addition, longer surgical time and surgeon experience have also been reported to be independently associated with the development of postoperative hypoxemia in the literature (23, 24). Therefore, more training and improved surgical skills of surgeons may benefit patients significantly.

The volume of intraoperative transfusion of RBCs was another independent risk factors for SH in our analysis results, which has also been reported previously (12, 13, 18). Although blood transfusion can be life-saving in cardiac surgery, increasing evidence suggests that massive transfusion of blood and blood products is associated with poorer clinical outcomes (25–34). Nakajima et al. found that the volume of transfused RBCs was positively associated with the risk of postoperative hypoxemia in patients undergoing surgery for acute type A aortic dissection, showing a dose-effect relationship (13). In the findings of Wang et al., blood transfusion more than 3,000 ml was identified as an independent risk factor for postoperative hypoxemia in Stanford A aortic dissection, associated with a 9.5-fold increased risk (18). The results of Sheng et al. demonstrated that blood transfusion more than 6 units was independently associated with postoperative hypoxemia and the risk increased to 12 times (12). Changes in immune function may partially explain the development of postoperative hypoxemia in response to blood transfusion (35, 36). The length of blood storage time can also have a certain impact on the development of postoperative hypoxemia due to the capacity to carry oxygen of the RBCs may decrease and the transfusion-related inflammation may increase with time (37, 38). On the other hand, massive blood transfusion may be due to massive bleeding, which can also have adverse consequences. Even so, current clinical guidelines on transfusion management still recommend the use of restrictive transfusion strategies in practice (39, 40).

Surgical types were identified as an independent risk factors and serum albumin was identified as a protective factor in the multivariate analysis. Compared with several other types of surgery, the risk of hypoxemia after aortic surgery was significantly higher. This was similar to several previous reports (6, 41, 42). In the results of Ge et al., the risk of postoperative hypoxemia was 4.5 times higher in patients undergoing Stanford type A aortic compared with type B (6). In the findings of Yoram, emergency surgery and hypoalbuminemia were identified as significant risk factors for post-CPB hypoxemia (41). Rady et al. obtained similar results, who identified that large BMI, hypoalbuminemia, emergency surgery, and prolonged CPB time were risk factors for the early onset of severe postoperative pulmonary dysfunction (41). The increased risk of aortic surgery may be related to greater surgical trauma and longer operative time. In our analysis, the overall discrimination was not significantly decreased after the removal of the variable of surgical type from the model 1, and the other factors were relatively more weighted instead. The albumin was similar, which meant that these two factors could be reflected and replaced by the other four predictors to some extent. Therefore, we excluded surgical types and albumin from the final model to construct a more parsimonious prediction model with good discrimination and calibration to facilitate clinical application.

Additionally, several other predictors for postoperative hypoxemia have also been reported previously in the literature but were not found to be significant when analyzed by the multivariate analysis in the current study, such as advanced age, hypertension, smoking history, renal insufficiency, female sex, poor cardiac function, and diabetes (1, 23, 24, 43, 44). Some postoperative factors have also been reported to be related to the occurrence of postoperative hypoxemia, but we did not include postoperative factors into our analysis due to these factors were not available early. Despite this, the final model we constructed performed well in various aspects.

The prediction model can be useful in predicting individual risk, identifying high-risk populations, and preventing adverse outcomes. Several prevention measures for hypoxemia and acute respiratory distress syndrome have been proposed in recent years, such as low tidal volume and inspiratory pressure ventilation, prone positioning, high-frequency oscillatory ventilation, lung recruitment maneuvers, appropriate application of positive end-expiratory pressure, and the use of extracorporeal membrane oxygenation (45). Using appropriate preventive interventions and treatments targeted at high-risk patients identified by the prediction model may result in substantial economic gains as well as improved clinical outcomes.

Several limitations were present in this study. First, this was a retrospective single-center study and was not validated outside, which may limit the model’s generalizability. Second, some factors, such as preoperative PaO_2_/FiO_2_, were not included in this study, which may be associated with the occurrence of SH. Third, the SH included in this study was within the first 24 h after surgery, which may underestimate the overall incidence of hypoxemia. Fourth, only the association between SH and in-hospital outcomes was analyzed, and long-term prognosis after discharge was not collected, which should be reinforced in the future.

## Conclusion

Severe hypoxemia after cardiac surgery was common and was related to poorer clinical outcomes. By using the multivariate analysis, six independent risk factors for SH after cardiac surgery were identified, such as surgical types, WBC, BMI, serum albumin, CPB time, and transfusion of RBCs. After comprehensive comparison and screening, a final model with excellent discriminative ability, calibration, and clinical utility was considered to be the most appropriate and parsimonious model, which included WBC, BMI, CPB time, and transfusion of RBCs. A nomogram and a web-based risk calculator was then established based on the final model, and three risk groups were divided. The prediction model may be helpful for informed decision-making, individualized risk assessment, high-risk patient identification, and targeted prevention.

## Data Availability Statement

The raw data supporting the conclusions of this article will be made available by the authors, without undue reservation.

## Ethics Statement

The studies involving human participants were reviewed and approved by the Ethics Committee of Tongji Medical College of Huazhong University of Science and Technology. Written informed consent for participation was not required for this study in accordance with the national legislation and the institutional requirements.

## Author Contributions

CL, FX, and FS: conception and design. XLD, ZY, and XH: administrative support. MS, PY, and DW: provision of study materials or patients. DW, XH, and YS: collection and assembly of data. XCD and DW: data analysis and interpretation. All authors: manuscript writing and final approval of manuscript.

## Conflict of Interest

The authors declare that the research was conducted in the absence of any commercial or financial relationships that could be construed as a potential conflict of interest. The reviewer SL declared a shared parent affiliation with the authors XCD and YS at the time of review.

## Publisher’s Note

All claims expressed in this article are solely those of the authors and do not necessarily represent those of their affiliated organizations, or those of the publisher, the editors and the reviewers. Any product that may be evaluated in this article, or claim that may be made by its manufacturer, is not guaranteed or endorsed by the publisher.

## Footnotes

1
www.R-project.org/

2
https://severehypoxemia.shinyapps.io/dynnomapp/

## Abbreviations

AUC: area under the receiver operating characteristic curve
BMI: body mass index
CI: confidence interval
CPB: cardiopulmonary bypass
ICU: intensive care unit
NYHA: New York Heart Association
OR: odds ratio
PaO_2_-FiO_2_: arterial oxygen tension-inspired oxygen concentration
RBC: red blood cell
ROC: receiver operating characteristic curve
SH: severe hypoxemia
WBC: white blood cell.

## References

[1] Zhou J Pan J Yu Y Huang W Lai Y Liang W Independent risk factors of hypoxemia in patients after surgery with acute type A aortic dissection. *Ann Palliat Med.* (2021) 10:7388–97. 10.21037/apm-21-1428 34263634

[2] Dunham AM Grega MA Brown CT McKhann GM Baumgartner WA Gottesman RF. Perioperative low arterial oxygenation is associated with increased stroke risk in cardiac surgery. *Anesth Analg.* (2017) 125:38–43. 10.1213/ANE.0000000000002157 28614129

[3] Ranucci M Ballotta A La Rovere MT Castelvecchio S. Postoperative hypoxia and length of intensive care unit stay after cardiac surgery: the underweight paradox? *PLos One.* (2014) 9:e93992. 10.1371/journal.pone.0093992 24709952 PMC3978074

[4] Wu Z Wang Z Wu H Hu R Ren W Hu Z Obesity is a risk factor for preoperative hypoxemia in Stanford A acute aortic dissection. *Medicine (Baltimore).* (2020) 99:e19186. 10.1097/MD.0000000000019186 32176045 PMC7440331

[5] Gong M Wu Z Xu S Li L Wang X Guan X Increased risk for the development of postoperative severe hypoxemia in obese women with acute type a aortic dissection. *J Cardiothorac Surg.* (2019) 14:81. 10.1186/s13019-019-0888-9 31023343 PMC6482483

[6] Ge H Jiang Y Jin Q Wan L Qian X Zhang Z. Nomogram for the prediction of postoperative hypoxemia in patients with acute aortic dissection. *BMC Anesthesiol.* (2018) 18:146. 10.1186/s12871-018-0612-7 30342471 PMC6195757

[7] Ranieri VM Rubenfeld GD Thompson BT Ferguson ND Caldwell E Fan E Acute respiratory distress syndrome: the Berlin Definition. *JAMA.* (2012) 307:2526–33. 10.1001/jama.2012.5669 22797452

[8] Liu N Zhang W Ma W Shang W Zheng J Sun L. Risk factors for hypoxemia following surgical repair of acute type A aortic dissection. *Interact Cardiovasc Thorac Surg.* (2017) 24:251–6. 10.1093/icvts/ivw272 27756811

[9] Shi S Gao Y Wang L Liu J Yuan Z Yu M. Elevated free fatty acid level is a risk factor for early postoperative hypoxemia after on-pump coronary artery bypass grafting: association with endothelial activation. *J Cardiothorac Surg.* (2015) 10:122. 10.1186/s13019-015-0323-9 26381483 PMC4574443

[10] Santos NP Mitsunaga RM Borges DL Costa MA Baldez TE Lima IM Factors associated to hypoxemia in patients undergoing coronary artery bypass grafting. *Rev Bras Cir Cardiovasc.* (2013) 28:364–70. 10.5935/1678-9741.20130056 24343686

[11] Szeles TF Yoshinaga EM Alenca W Brudniewski M Ferreira FS Auler JOCJ Hypoxemia after myocardial revascularization: analysis of risk factors. *Rev Bras Anestesiol.* (2008) 58:124–36. 10.1590/s0034-70942008000200005 19378531

[12] Sheng W Yang H Chi Y Niu Z Lin M Long S. Independent risk factors for hypoxemia after surgery for acute aortic dissection. *Saudi Med J.* (2015) 36:940–6. 10.15537/smj.2015.8.11583 26219444 PMC4549590

[13] Nakajima T Kawazoe K Izumoto H Kataoka T Niinuma H Shirahashi N. Risk factors for hypoxemia after surgery for acute Type A aortic dissection. *Surg Today (Tokyo, Japan).* (2006) 36:680–5. 10.1007/s00595-006-3226-5 16865510

[14] Hibbert K Rice M Malhotra A. Obesity and ARDS. *Chest.* (2012) 142:785–90. 10.1378/chest.12-0117 22948584 PMC3435141

[15] Duan XZ Xu ZY Lu FL Han L Tang YF Tang H Inflammation is related to preoperative hypoxemia in patients with acute Stanford type A aortic dissection. *J Thorac Dis.* (2018) 10:1628–34. 10.21037/jtd.2018.03.48 29707315 PMC5906234

[16] Kumar S Payal N Srivastava VK Kaushik S Saxena J Jyoti A. Neutrophil extracellular traps and organ dysfunction in sepsis. *Clin Chim Acta.* (2021) 523:152–62. 10.1016/j.cca.2021.09.012 34537216

[17] Colgan SP Furuta GT Taylor CT. Hypoxia and innate immunity: keeping up with the HIFsters. *Annu Rev Immunol.* (2020) 38:341–63. 10.1146/annurev-immunol-100819-121537 31961750 PMC7924528

[18] Wang Y Xue S Zhu H. Risk factors for postoperative hypoxemia in patients undergoing Stanford A aortic dissection surgery. *J Cardiothorac Surg.* (2013) 8:118. 10.1186/1749-8090-8-118 23631417 PMC3649943

[19] Raja SG Dreyfus GD. Modulation of systemic inflammatory response after cardiac surgery. *Asian Cardiovasc Thorac Ann.* (2005) 13:382–95. 10.1177/021849230501300422 16304234

[20] Asimakopoulos G. Systemic inflammation and cardiac surgery: an update. *Perfusion.* (2001) 16:353–60. 10.1177/026765910101600505 11565890

[21] Anastasiadis K Antonitsis P Deliopoulos A Argiriadou H. A multidisciplinary perioperative strategy for attaining “more physiologic” cardiac surgery. *Perfusion.* (2017) 32:446–53. 10.1177/0267659117700488 28692337

[22] Anastasiadis K Argiriadou H Deliopoulos A Antonitsis P. Minimal invasive extracorporeal circulation (MiECC): the state-of-the-art in perfusion. *J Thorac Dis.* (2019) 11:S1507–14. 10.21037/jtd.2019.01.66 31293801 PMC6586580

[23] Liu TMP Shih MM Lee WMP Wang KM Liu C Hung CM Hypoxemia during one-lung ventilation for robot-assisted coronary artery bypass graft surgery. *Ann Thoracic Surg.* (2013) 96:127–32. 10.1016/j.athoracsur.2013.04.017 23731612

[24] Lan L Cen Y Jiang L Miao H Lu W. Risk factors for the development of intraoperative hypoxia in patients undergoing nonintubated video-assisted thoracic surgery: a retrospective study from a single center. *Med Sci Monit.* (2021) 27:e928965. 10.12659/MSM.928965 33901163 PMC8086517

[25] Sultan I Bianco V Aranda-Michel E Kilic A Serna-Gallegos D Navid F The use of blood and blood products in aortic surgery is associated with adverse outcomes. *J Thoracic Cardiovasc Surg.* (2021):S0022–5223. [Online ahead of print], 10.1016/j.jtcvs.2021.02.096 33838909

[26] Crawford TC Magruder JT Fraser C Suarez-Pierre A Alejo D Bobbitt J Less is more: results of a statewide analysis of the impact of blood transfusion on coronary artery bypass grafting outcomes. *Ann Thorac Surg.* (2018) 105:129–36. 10.1016/j.athoracsur.2017.06.062 29074154

[27] Wang D Le S Wu J Xie F Li X Wang H Nomogram for postoperative headache in adult patients undergoing elective cardiac surgery. *J Am Heart Assoc.* (2022) 11:e023837. 10.1161/JAHA.121.023837 35411784 PMC9238448

[28] Wang D Wang S Song Y Wang H Zhang A Wu L Predictors and outcomes of postoperative tracheostomy in patients undergoing acute type A aortic dissection surgery. *BMC Cardiovasc Disord.* (2022) 22:94. 10.1186/s12872-022-02538-4 35264113 PMC8908588

[29] Wang D Li Y Sheng W Wang H Le S Huang X Development and validation of a nomogram model for pneumonia after redo cardiac surgery. *J Cardiovasc Med.* (2022): [Online ahead of print], 10.2459/JCM.0000000000001302 37594436

[30] Wang D Abuduaini X Huang X Wang H Chen X Le S Development and validation of a risk prediction model for postoperative pneumonia in adult patients undergoing Stanford type A acute aortic dissection surgery: a case control study. *J Cardiothorac Surg.* (2022) 17:22. 10.1186/s13019-022-01769-y 35197097 PMC8864916

[31] Wang D Le S Luo J Chen X Li R Wu J Incidence, risk factors and outcomes of postoperative headache after stanford Type A acute aortic dissection surgery. *Front Cardiovasc Med.* (2021) 8:781137. 10.3389/fcvm.2021.781137 35004895 PMC8733002

[32] Wang D Wang S Wu J Le S Xie F Li X Nomogram models to predict postoperative hyperlactatemia in patients undergoing elective cardiac surgery. *Front Med.* (2021) 8:763931. 10.3389/fmed.2021.763931 34926506 PMC8674505

[33] Wang D Huang X Wang H Le S Du X. Clinical risk score for postoperative pneumonia following heart valve surgery. *Chin Med J Peking.* (2021) 134:2447–56. 10.1097/CM9.0000000000001715 34669637 PMC8654438

[34] Wang D Chen X Wu J Le S Xie F Li X Development and validation of nomogram models for postoperative pneumonia in adult patients undergoing elective cardiac surgery. *Front Cardiovasc Med.* (2021) 8:750828. 10.3389/fcvm.2021.750828 34708096 PMC8542719

[35] Karsten E Herbert BR. The emerging role of red blood cells in cytokine signalling and modulating immune cells. *Blood Rev.* (2020) 41:100644. 10.1016/j.blre.2019.100644 31812320

[36] Tormey CA Hendrickson JE. Transfusion-related red blood cell alloantibodies: induction and consequences. *Blood.* (2019) 133:1821–30. 10.1182/blood-2018-08-833962 30808636 PMC6484385

[37] Hod EA Brittenham GM Billote GB Francis RO Ginzburg YZ Hendrickson JE Transfusion of human volunteers with older, stored red blood cells produces extravascular hemolysis and circulating non-transferrin-bound iron. *Blood.* (2011) 118:6675–82. 10.1182/blood-2011-08-371849 22021369 PMC3242722

[38] Roback JD Neuman RB Quyyumi A Sutliff R. Insufficient nitric oxide bioavailability: a hypothesis to explain adverse effects of red blood cell transfusion. *Transfusion.* (2011) 51:859–66. 10.1111/j.1537-2995.2011.03094.x 21496047 PMC4793902

[39] Carson JL Guyatt G Heddle NM Grossman BJ Cohn CS Fung MK Clinical practice guidelines from the AABB: red blood cell transfusion thresholds and storage. *JAMA.* (2016) 316:2025–35. 10.1001/jama.2016.9185 27732721

[40] Jeffrey L Apfelbaum MD Gregory A Nuttall MD Richard T Connis PD Practice guidelines for perioperative blood management: an updated report by the American Society of Anesthesiologists Task Force on Perioperative Blood Management*. *Anesthesiology.* (2015) 122:241–75. 10.1097/ALN.0000000000000463 25545654

[41] Weiss YG Merin G Koganov E Ribo A Oppenheim-Eden A Medalion B Postcardiopulmonary bypass hypoxemia: a prospective study on incidence, risk factors, and clinical significance. *J Cardiothorac Vasc Anesth.* (2000) 14:506–13. 10.1053/jcan.2000.9488 11052429

[42] Rady MY Ryan T Starr NJ. Early onset of acute pulmonary dysfunction after cardiovascular surgery: risk factors and clinical outcome. *Crit Care Med.* (1997) 25:1831–9. 10.1097/00003246-199711000-00021 9366766

[43] Sheng W Le S Song Y Du Y Wu J Tang C Preoperative nomogram and risk calculator for postoperative hypoxemia and related clinical outcomes following stanford Type A acute aortic dissection surgery. *Front Cardiovasc Med.* (2022) 9:851447. 10.3389/fcvm.2022.851447 35548419 PMC9082545

[44] Ji Q Mei Y Wang X Feng J Cai J Sun Y Study on the risk factors of postoperative hypoxemia in patients undergoing coronary artery bypass grafting. *Circ J Off J Japanese Circ Soc.* (2008) 72:1975–80. 10.1253/circj.CJ-08-0369 18931449

[45] Fan E Brodie D Slutsky AS. Acute respiratory distress syndrome: advances in diagnosis and treatment. *JAMA J Am Med Assoc.* (2018) 319:698–710. 10.1001/jama.2017.21907 29466596

