# Supplementary material for: Incidence, Risk Factors, and Outcomes of Severe Hypoxemia After Cardiac Surgery
Source: Front Cardiovasc Med. 2022 Jun 28;9:934533. doi: 10.3389/fcvm.2022.934533 (PMC9273816; doi:10.3389/fcvm.2022.934533)
Supplement: Supplementary file 1 [file Table_1.DOCX]

Supplementary table 1. Multivariate analysis of independent risk factors for SH after cardiac surgery (model 1)

| Characteristics | Coefficient | OR (95% CI) | *P* value |
| --- | --- | --- | --- |
| Surgical Types |  |  | <0.001 |
| Isolated valve surgery | Reference | Reference | Reference |
| Isolated coronary artery bypass grafting | 0.010 | 1.010 (0.501-2.040) | 0.977 |
| Mixed valve and coronary artery bypass grafting | 0.145 | 1.156 (0.589-2.272) | 0.674 |
| Aortic surgery | 1.080 | 2.944 (1.831-4.732) | <0.001 |
| Other types | -0.008 | 0.992 (0.344-2.865) | 0.989 |
| White blood cell count (× 109/L) | 0.143 | 1.153 (1.102-1.207) | <0.001 |
| Body mass index (kg/m2) | 0.241 | 1.273 (1.215-1.334) | <0.001 |
| Serum albumin (g/L) | -0.054 | 0.948 (0.913-0.984) | 0.005 |
| Cardiopulmonary bypass time (minutes) | 0.006 | 1.006 (1.003-1.008) | <0.001 |
| Transfusion of red blood cells (units) | 0.158 | 1.171 (1.088-1.262) | <0.001 |
| Constant | -9.938 | <0.001 | <0.001 |

Abbreviations: CI, confidence interval; OR, odds ratio; SH, severe hypoxemia.Supplementary table 2. The results of multivariate analysis of independent risk factors for SH after the exclusion of surgical types on the basis of model 1 (model 2)

| Characteristics | Coefficient | OR (95% CI) | *P* value |
| --- | --- | --- | --- |
| White blood cell count (× 109/L) | 0.185 | 1.203 (1.153-1.254) | <0.001 |
| Body mass index (kg/m2) | 0.252 | 1.287 (1.229-1.348) | <0.001 |
| Serum albumin (g/L) | -0.062 | 0.940 (0.905-0.975) | 0.001 |
| Cardiopulmonary bypass time (minutes) | 0.008 | 1.008 (1.006-1.010) | <0.001 |
| Transfusion of red blood cells (units) | 0.191 | 1.211 (1.130-1.298) | <0.001 |
| Constant | -10.183 | <0.001 | <0.001 |

Abbreviations: CI, confidence interval; OR, odds ratio; SH, severe hypoxemia.Supplementary table 3. The results of multivariate analysis of independent risk factors for SH after the exclusion of white blood cell count on the basis of model 2 (model 3)

| Characteristics | Coefficient | OR (95% CI) | *P* value |
| --- | --- | --- | --- |
| Body mass index (kg/m2) | 0.285 | 1.330 (1.273-1.389) | <0.001 |
| Serum albumin (g/L) | -0.074 | 0.928 (0.895-0.963) | <0.001 |
| Cardiopulmonary bypass time (minutes) | 0.010 | 1.010 (1.008-1.013) | <0.001 |
| Transfusion of red blood cells (units) | 0.219 | 1.245 (1.166-1.329) | <0.001 |
| Constant | -9.588 | <0.001 | <0.001 |

Abbreviations: CI, confidence interval; OR, odds ratio; SH, severe hypoxemia.Supplementary table 4. The results of multivariate analysis of independent risk factors for SH after the exclusion of body mass index on the basis of model 2 (model 4)

| Characteristics | Coefficient | OR (95% CI) | *P* value |
| --- | --- | --- | --- |
| White blood cell count (× 109/L) | 0.229 | 1.257 (1.208-1.308) | <0.001 |
| Serum albumin (g/L) | -0.024 | 0.976 (0.943-0.999) | 0.044 |
| Cardiopulmonary bypass time (minutes) | 0.009 | 1.009 (1.007-1.011) | <0.001 |
| Transfusion of red blood cells (units) | 0.169 | 1.184 (1.107-1.267) | <0.001 |
| Constant | -5.793 | 0.003 | <0.001 |

Abbreviations: CI, confidence interval; OR, odds ratio; SH, severe hypoxemia.Supplementary table 5. The results of multivariate analysis of independent risk factors for SH after the exclusion of serum albumin on the basis of model 2 (model 5)

| Characteristics | Coefficient | OR (95% CI) | *P* value |
| --- | --- | --- | --- |
| White blood cell count (× 109/L) | 0.191 | 1.211 (1.160-1.263) | <0.001 |
| Body mass index (kg/m2) | 0.240 | 1.271 (1.215-1.330) | <0.001 |
| Cardiopulmonary bypass time (minutes) | 0.008 | 1.008 (1.006-1.010) | <0.001 |
| Transfusion of red blood cells (units) | 0.210 | 1.234 (1.152-1.321) | <0.001 |
| Constant | -12.448 | <0.001 | <0.001 |

Abbreviations: CI, confidence interval; OR, odds ratio; SH, severe hypoxemia.Supplementary table 6. The results of multivariate analysis of independent risk factors for SH after the exclusion of cardiopulmonary bypass time on the basis of model 2 (model 6)

| Characteristics | Coefficient | OR (95% CI) | *P* value |
| --- | --- | --- | --- |
| White blood cell count (× 109/L) | 0.224 | 1.251 (1.201-1.302) | <0.001 |
| Body mass index (kg/m2) | 0.262 | 1.299 (1.242-1.359) | <0.001 |
| Serum albumin (g/L) | -0.066 | 0.936 (0.902-0.971) | <0.001 |
| Transfusion of red blood cells (units) | 0.303 | 1.353 (1.276-1.435) | <0.001 |
| Constant | -9.731 | <0.001 | <0.001 |

Abbreviations: CI, confidence interval; OR, odds ratio; SH, severe hypoxemia.Supplementary table 7. The results of multivariate analysis of independent risk factors for SH after the exclusion of transfusion of red blood cells on the basis of model 2 (model 7)

| Characteristics | Coefficient | OR (95% CI) | *P* value |
| --- | --- | --- | --- |
| White blood cell count (× 109/L) | 0.202 | 1.223 (1.173-1.276) | <0.001 |
| Body mass index (kg/m2) | 0.244 | 1.276 (1.220-1.335) | <0.001 |
| Serum albumin (g/L) | -0.079 | 0.924 (0.891-0.959) | <0.001 |
| Cardiopulmonary bypass time (minutes) | 0.011 | 1.011 (1.009-1.013) | <0.001 |
| Constant | -9.305 | <0.001 | <0.001 |

Abbreviations: CI, confidence interval; OR, odds ratio; SH, severe hypoxemia.Supplementary table 8. The results of multivariate analysis of independent risk factors for SH after the exclusion of white blood cell count on the basis of model 5 (model 8)

| Characteristics | Coefficient | OR (95% CI) | *P* value |
| --- | --- | --- | --- |
| Body mass index (kg/m2) | 0.274 | 1.315 (1.259-1.373) | <0.001 |
| Cardiopulmonary bypass time (minutes) | 0.011 | 1.011 (1.009-1.013) | <0.001 |
| Transfusion of red blood cells (units) | 0.239 | 1.270 (1.191-1.354) | <0.001 |
| Constant | -12.346 | <0.001 | <0.001 |

Abbreviations: CI, confidence interval; OR, odds ratio; SH, severe hypoxemia.Supplementary table 9. The results of multivariate analysis of independent risk factors for SH after the exclusion of body mass index on the basis of model 5 (model 9)

| Characteristics | Coefficient | OR (95% CI) | *P* value |
| --- | --- | --- | --- |
| White blood cell count (× 109/L) | 0.232 | 1.261 (1.212-1.312) | <0.001 |
| Cardiopulmonary bypass time (minutes) | 0.009 | 1.009 (1.007-1.011) | <0.001 |
| Transfusion of red blood cells (units) | 0.178 | 1.195 (1.119-1.277) | <0.001 |
| Constant | -6.811 | 0.001 | <0.001 |

Abbreviations: CI, confidence interval; OR, odds ratio; SH, severe hypoxemia.Supplementary table 10. The results of multivariate analysis of independent risk factors for SH after the exclusion of cardiopulmonary bypass time on the basis of model 5 (model 10)

| Characteristics | Coefficient | OR (95% CI) | *P* value |
| --- | --- | --- | --- |
| White blood cell count (× 109/L) | 0.233 | 1.263 (1.213-1.315) | <0.001 |
| Body mass index (kg/m2) | 0.248 | 1.281 (1.226-1.339) | <0.001 |
| Transfusion of red blood cells (units) | 0.324 | 1.383 (1.305-1.464) | <0.001 |
| Constant | -12.130 | <0.001 | <0.001 |

Abbreviations: CI, confidence interval; OR, odds ratio; SH, severe hypoxemia.Supplementary table 11. The results of multivariate analysis of independent risk factors for SH after the exclusion of transfusion of red blood cells on the basis of model 5 (model 11)

| Characteristics | Coefficient | OR (95% CI) | *P* value |
| --- | --- | --- | --- |
| White blood cell count (× 109/L) | 0.212 | 1.236 (1.184-1.290) | <0.001 |
| Body mass index (kg/m2) | 0.228 | 1.256 (1.201-1.312) | <0.001 |
| Cardiopulmonary bypass time (minutes) | 0.012 | 1.012 (1.010-1.014) | <0.001 |
| Constant | -12.163 | <0.001 | <0.001 |

Abbreviations: CI, confidence interval; OR, odds ratio; SH, severe hypoxemia.
